# Associations among structural brain alterations, respiratory symptoms and cognitive impairment in patients with chronic obstructive pulmonary disease-related subclinical cognitive vulnerability: a multimodal neuroimaging study

**DOI:** 10.3389/fnagi.2026.1797616

**Published:** 2026-05-18

**Authors:** Chunrong Wang, Shiwei Lin, Kun Zhao, Yafeng Zhan, Yingwei Qiu, Kewen Peng, Xinyu Wang, Su Lui, Yong Liu, Shijun Qiu

**Affiliations:** 1 Department of Radiology, Shenzhen Nanshan People’s Hospital, Shenzhen, Guangdong, China; 2 Department of Radiology, Affiliated Nanshan Hospital of Shenzhen University, Shenzhen, Guangdong, China; 3 School of Artificial Intelligence, Beijing University of Posts and Telecommunications, Beijing, China; 4 School of Biomedical Engineering, Southern Medical University, Guangzhou, China; 5 Department of Radiology, Huaxi MR Research Center (HMRRC), Institute of Radiology and Medical Imaging, West China Hospital of Sichuan University, Chengdu, Sichuan, China; 6 Psychoradiology Key Laboratory of Sichuan Province, West China Hospital of Sichuan University, Chengdu, Sichuan, China; 7 School of Artificial Intelligence, University of Chinese Academy of Sciences & Brainnetome Center, Chinese Academy of Sciences, Beijing, China; 8 Department of Radiology, First Affiliated Hospital of Guangzhou University of Chinese Medicine, Guangzhou, Guangdong, China; 9 State Key Laboratory of Traditional Chinese Medicine Syndrome, Guangzhou, Guangdong, China

**Keywords:** chronic obstructive pulmonary disease, cognitive impairment, fractional anisotropy, multimodal magnetic resonance imaging, radial diffusivity, structural network

## Abstract

**Background:**

Chronic obstructive pulmonary disease (COPD) is a systemic condition that often results in cognitive impairment. Nevertheless, the central nervous system correlates of COPD-related cognitive decline are not well-defined, representing a critical knowledge gap. The present study aimed to investigate the relationship among respiratory symptoms, structural brain changes, and cognitive impairment in patients with COPD who have subclinical cognitive vulnerability (COPD-SCV).

**Methods:**

This cross-sectional study included 60 patients with stable COPD-SCV and 60 age-, sex-, and education-matched controls. Multimodal magnetic resonance imaging was used to measure cortical thickness, fractional anisotropy (FA), radial diffusivity (RD), and graph-theoretical network metrics. Two tests were used to evaluate cognitive performance: the Mini-Mental State Examination (MMSE) and the Montreal Cognitive Assessment (MoCA). The St. George’s Respiratory Questionnaire (SGRQ) was used to estimate respiratory symptoms. To investigate the relationship among respiratory symptoms, structural brain alterations, and cognitive impairment, we conducted correlation and mediation analyses.

**Results:**

MoCA scores, used as a screening tool, indicated lower cognitive performance in patients with COPD than in healthy controls (25.03 ± 3.13 vs. 27.13 ± 2.32; *p* < 0.001), with preliminary observations suggesting potential involvement in memory and visuospatial/executive domains. These deficiencies were accompanied by cortical thinning in the right cuneus, temporal lobe, and supplementary motor area (SMA; *p* < 0.05), along with white matter disruption (lower FA, higher RD) in corresponding tracts (*p* < 0.01). Graphical analysis showed decreased degree centrality of the left SMA (SMA_L’DC, *p* = 0.005) and decreased global network efficiency (*p* < 0.001). Notably, SMA_L’DC was significantly associated with respiratory symptoms (SGRQ) and cognitive performance (MoCA). Using a cross-sectional mediation model, we found that the association between SGRQ and MoCA was significantly accounted for by SMA_L’DC (path c: *β* = −0.302, *p* = 0.019). Specifically, the results supported a statistical mediation model in which SMA_L’DC was significantly associated with the pathway linking respiratory symptoms and cognitive function.

**Conclusion:**

Patients with COPD-SCV exhibit deterioration of grey matter morphology, white matter microstructure, and structural network. The relationship between respiratory symptoms and cognitive impairment shows a statistical association with variations in SMA_L connectivity, a key network hub. These findings identify a distinct neuroanatomical phenotype and suggest that SMA connectivity may be a potential imaging marker associated with cognitive risk. However, given the cross-sectional design, these results reflect correlational patterns consistent with a lung–brain axis hypothesis rather than definitive evidence of causation.

## 1 Introduction

Chronic obstructive pulmonary disease (COPD) is a systemic disorder characterized by the hallmark feature of persistent airflow limitation (Ding et al., 2025; Zhang et al., 2025). Cognitive impairment, a prevalent and serious extra-pulmonary comorbidity of COPD, is linked to increased mortality, poor treatment compliance, and increased use of medical resources (Dodd et al., 2012; Cleutjens et al., 2017; Hao and Bu, 2025). Approximately 56.7% of patients with stable COPD have cognitive impairment, and its prevalence is four-fold higher than in individuals without COPD (Ding et al., 2025; Zhang et al., 2025). Unlike Alzheimer’s disease (Cleutjens et al., 2017), mild cognitive impairment (MCI) associated with COPD is mostly non-amnestic and manifests as impairments in verbal memory, attention, and linguistic fluency (Morris et al., 2019). In COPD, cognitive decline generally progresses insidiously from MCI to dementia in an insidious manner.

A critical but often ignored window for avoiding dementia is the subclinical cognitive vulnerability (SCV) phase, in which cognitive deterioration is asymptomatic (Rosso et al., 2023). This stage predates the development of MCI and is characterized by mild neuropsychological abnormalities that are challenging to detect with conventional cognitive tests, making diagnosis challenging. The lack of this subclinical COPD population is further complicated by the absence of defined diagnostic criteria. Although MCI is considered a potentially reversible state and represents a critical window for intervention (Siraj, 2023; Kotani et al., 2025; Shimada et al., 2019), early and accurate diagnosis is crucial. Indeed, interventions such as pulmonary rehabilitation improved cognitive function in some patients with COPD. However, evidence remains heterogeneous, highlighting the need for early characterization of at-risk individuals (Dibajnia et al., 2026).

To explore the earliest “asymptomatic” stage of cognitive decline in COPD, we introduced an operational study label termed COPD-related subclinical cognitive vulnerability (COPD-SCV). This label characterizes patients with preserved Mini-Mental State Examination (MMSE) scores but abnormal or subtly lower cognition, as assessed by the Montreal Cognitive Assessment (MoCA), indicating a stage preceding the onset of overt cognitive impairment. The MMSE is frequently used for clinical cognitive screening but has limited sensitivity for detecting mild impairments that can be identified by the MoCA (Rosso et al., 2023; Ramesh et al., 2024). Importantly, recent longitudinal studies suggest that early cognitive deficits in COPD are potentially reversible with targeted interventions such as pulmonary rehabilitation and anti-inflammatory treatment (Ryu et al., 2013; Chen et al., 2016). The majority of earlier research, which frequently involved patients with severe hypoxemia or advanced disease, focused on obvious MCI or dementia as detected by the MMSE or MoCA. In this study, we examine the initial stage of subclinical cognitive impairment in individuals with normal MMSE scores. Early detection of brain abnormalities may improve clinical outcomes, enable prompt management, and lessen the financial burden on patients. Our study cohort is defined as follows: patients with COPD diagnosed through medical history screening and physical examination with unimpaired activities of daily living (ADL) and objective neuropsychological tests showing deviations in cognitive function from baseline or within the lower end of the normal range (MMSE ≥ 27 points). Notably, these patients did not meet the diagnostic criteria for MCI or dementia, with no subjective cognitive complaints reported by the patients or their family members, and no functional impairments were observed.

Multimodal magnetic resonance imaging (MRI) can be used to non-invasively examine COPD-related brain changes. Recent systematic reviews have demonstrated that multimodal MRI (combining cortical morphometry, diffusion tensor imaging [DTI], and functional connectivity) provides a more comprehensive and sensitive detection of early microstructural alterations in COPD than single-modality approaches (Aging Biomarker et al., 2023). Widespread cerebral changes in patients with COPD, including gray matter (GM) atrophy, loss of white matter (WM) integrity, and reduced network efficiency, have been demonstrated in patients with COPD (Li and Fei, 2013; Zhang H. et al., 2013; Wang et al., 2017). For example, Zhang H. et al. (2013) observed significant cortical thinning in patients with COPD with hypoxemia, Lazari et al. (2021) found WM damage, and Wang et al. (2020) revealed abnormalities in default mode network connectivity. Previous neuroimaging studies in patients with COPD have utilized single-modality methods (e.g., voxel-based morphometry), which produced inconsistent results due to methodological limitations (Wang et al., 2017; Yin et al., 2019). Owing to the various limitations of single-modality MRI studies, the primary brain mechanisms of cognitive impairment in patients with COPD remain incompletely understood. Although functional MRI studies have reported connectivity disruptions in patients with COPD (Yu et al., 2016; Hu et al., 2018), graph-theoretical analyses of WM networks using DTI remain limited, despite their successful application in other conditions such as heart failure or thyroid-related disorders (Wang et al., 2023; Wu et al., 2021). The precise neural pathways connecting respiratory symptoms to cognitive decline remain unclear, even though recent research, such as the association between frailty and cortical thickness reported by Fukatsu-Chikumoto et al. (2024) implicates cortical thinning and WM damage. Particularly, in patients with normal global cognitive function, the mediating function of important network centers, including the supplementary motor area (SMA), in this relationship has not been sufficiently investigated.

Recent developments in multimodal neuroimaging, including high-resolution cortical morphometry, DTI, and graph-theoretical network analysis have enabled a more comprehensive approach of investigating COPD (Meng et al., 2017; Yuan et al., 2025). These techniques allow the simultaneous evaluation of cortical thinning, WM integrity, and network connectivity, offering an integrated perspective on neurobiological changes that surpasses that of single-modality imaging methods. Systematic reviews have consistently shown that combining data from multiple modalities (e.g., structural MRI, DTI, positron emissions tomography [PET]) improves diagnostic and prognostic accuracy for cognitive impairment compared with any single modality alone, providing complementary information on different aspects of neuropathology (Huang et al., 2023). However, the application of multiple modalities in COPD remains limited and has largely focused on individuals with clear cognitive impairment. Dodd et al. (2012) identified WM deficits in patients with COPD using DTI and resting-state functional MRI. Wang et al. (2020) reported a correlation between cognitive impairment and decreased GM volume and functional connectivity in patients with stable COPD. Yin et al. (2019) used a multimodal approach to demonstrate progressive structural brain abnormalities in patients with COPD caused by pulmonary function severity and highlighted the need for prompt intervention. Notably, slight alterations and functional brain changes may already be present before clinically significant cognitive dysfunction becomes apparent. Identifying this transition from compensation to decompensation is essential for the timely detection and management of cognitive decline. Nevertheless, few multimodal MRI studies have specifically examined patients with COPD-SCV.

To address these gaps, we compared patients with COPD-SCV and matched healthy controls (HCs) to investigate the relationships among respiratory symptoms, cognitive impairment, and structural brain abnormalities. We hypothesized that cortical thinning, WM microstructural disruption, and reduced network efficiency are present in patients with COPD-SCV. Furthermore, we aimed to determine whether structural brain changes are related to the cross-sectional association between respiratory symptoms and cognitive impairment. This would reflect a potential indirect pathway consistent with the lung–brain axis hypothesis, a concept describing the complex bidirectional communication network between the respiratory and central nervous systems (Guo et al., 2025; Yu et al., 2025; Huang et al., 2025).

## 2 Materials and methods

### 2.1 Ethical approval

This study was approved by the Medical Ethics Committee of Shenzhen Nanshan People’s Hospital. All procedures followed the principles outlined in the Declaration of Helsinki. Each participant provided written informed consent prior to enrollment in the clinical assessment and imaging protocols.

### 2.2 Participants

The study comprised 60 patients diagnosed with stable COPD-SCV and 60 demographically matched healthy normal controls (NCs), enrolled between July 2012 and May 2015. Patients with COPD were recruited from the inpatient wards and outpatient respiratory clinic of Shenzhen Nanshan People’s Hospital. Controls were recruited from the neighborhood, local communities, the physical examination department of Shenzhen Nanshan People’s Hospital, or from healthy volunteers. Age, sex, body mass index (BMI), and years of education were matched between groups. Systematic health questionnaire surveys confirmed that NCs had no history of progressive dyspnea, chronic cough, sputum production, or other respiratory symptoms. Participants with any underlying conditions related to respiratory or cognitive dysfunction were excluded.

All enrolled patients were in a clinically stable state, defined as the absence of acute exacerbations for at least 6 months prior to enrollment. Acute exacerbation was defined as a sustained worsening of respiratory symptoms (dyspnea, cough, or sputum production) beyond normal daily variations that required a change in medication or hospitalization (Vestbo et al., 2012; MacIntyre and Huang, 2008; Rodriguez-Roisin, 2000; Donaldson and Wedzicha, 2014).

To investigate the earliest stages of cognitive decline in COPD, we recruited a cohort representing the COPD-SCV spectrum, as defined below.

Inclusion Criteria (Defining COPD-SCV as an Operational Study Label):

(1) COPD diagnosis: Patients were diagnosed according to the Global Initiative for Chronic Obstructive Lung Disease (GOLD) criteria (Abdool-Gaffar et al., 2011). Airflow limitation was defined as a post-bronchodilator forced expiratory volume in 1 s/forced vital capacity (FEV_1_/FVC) ratio <0.70, confirmed by spirometry.

Disease severity stratification: Patients were classified into GOLD stages based on post-bronchodilator FEV_1_ (%predicted):

GOLD 1 (mild): FEV_1_ ≥ 80% predicted.
GOLD 2 (moderate): 50% ≤ FEV_1_ < 80% predicted.
GOLD 3 (severe): 30% ≤ FEV_1_ < 50% predicted.
GOLD 4 (very severe): FEV_1_ < 30% predicted.

(2) Preserved global cognition: An MMSE score ≥27 was used to exclude patients with overt dementia or moderate cognitive impairment (Folstein et al., 1975; Mitchell, 2009), consistent with the operational definition of COPD-SCV developed in this study.
(3) Absence of subjective complaints: No cognitive complaints reported by the patient or their family members during history taking, aligning with the asymptomatic nature of COPD-SCV defined in this study.
(4) Functional independence: Preserved ADL, ruling out the functional impairment required for an MCI diagnosis, as per the operational definition of COPD-SCV used in this study.

### 2.3 Clinical and neuropsychological assessment

Clinical and demographic data were systematically collected through medical record review and structured interviews. All cognitive and clinical assessments were completed prior to MRI. A board-certified neurologist performed a comprehensive neurological evaluation for each patient with COPD. The St. George’s Respiratory Questionnaire (SGRQ), which evaluates psychosocial impact, functional ability, and symptom burden, was used to assess respiratory health. All assessments were conducted by trained clinical personnel.

Physiological Protocol and Group Differences: Blood pressure, pulse rate, and arterial blood gas (ABG) analysis were included in the physiological assessment. Data collection differed between groups given ethical considerations regarding invasive procedures in healthy volunteers:

COPD Group: As per the inclusion criteria, all patients underwent spirometry for diagnostic confirmation prior to enrollment. On the day of MRI scanning, ABG analysis and spirometry were performed to assess current respiratory status.

Control Group (NCs): In accordance with ethical guidelines minimizing invasive procedures, ABG analysis and spirometry were not performed on the NCs. Respiratory health status was assessed using structured questionnaires and exclusion criteria (i.e., absence of dyspnea, chronic cough, or sputum production). Although this indicates that NCs were free of respiratory symptoms, the lack of objective spirometry or ABG data represents a study limitation (addressed in the Discussion).

The neuropsychological assessment included several instruments. The Chinese version of the MMSE was used to screen for general cognitive impairment. The Chinese version of the MoCA was used to detect subtle deficits in visuospatial ability, naming, attention, language, abstraction, memory, and orientation. The MoCA was specifically selected for its higher sensitivity in detecting mild cognitive impairment compared with the MMSE. To account for the effect of education on cognitive performance, 1 point was added to the total MoCA score for participants with ≤ 12 years of formal education.

#### 2.3.1 Exclusion criteria

Both groups were subject to strict exclusion criteria, including a history of pulmonary disease other than COPD; neurological disorders (e.g., cerebral hemorrhage, stroke, brain tumor, clinically diagnosed dementia, epilepsy, or head injury); However, we acknowledge that relying solely on clinical history to exclude neurodegenerative disorders does not account for preclinical or prodromal stages, particularly given the age of our cohort. Future studies should incorporate biomarker confirmation (e.g., cerebrospinal fluid [CSF] analysis for amyloid and tau proteins, PET imaging) to more definitively exclude these conditions. Systemic diseases known to affect cognition (e.g., hypertension, diabetes, congestive heart failure, hepatic failure, cirrhosis, malignancy, obstructive sleep apnea, anemia, or active psychiatric disorders) were also used as an exclusion criterion, and patients with a history of drug or alcohol abuse were excluded as well. Additional exclusion criteria were contraindications to MRI, such as ferromagnetic implants, pacemakers, or claustrophobia, as well as an MMSE score < 26. Furthermore, control participants with evidence of cognitive impairment or respiratory symptoms were excluded.

### 2.4 Methods of imaging

A Siemens MAGNETOM Skyra 3 T scanner (Siemens Healthcare, Erlangen, Germany) with a 32-channel phased-array head coil was used to collect all imaging data. To ensure data quality, we performed regular system calibrations and daily phantom imaging to monitor gradient stability, signal uniformity, and geometric accuracy.

#### 2.4.1 Structural MRI

A rapid acquisition gradient echo sequence was used to obtain high-resolution 3D T1-weighted images. The key imaging parameters were: repetition time, 2,250 ms; echo time, 2.19 ms; inversion time, 900 ms; flip angle, 9°; field of view, 256 × 256 mm^2^; matrix size, 256 × 256; isotropic voxel size, 1 mm^3^.

#### 2.4.2 DTI

A single-shot echo-planar imaging sequence was used to obtain diffusion-weighted images, with slices aligned with the anterior commissure–posterior commissure plane. The imaging parameters were: repetition time, 11,400 ms; echo time, 84 ms; b-values, 0 and 1,000 s/mm^2^; 64 diffusion gradient directions; 2-mm contiguous slices; matrix size, 128 × 128; field of view, 230 × 230 mm^2^. The acquisition lasted approximately 13 min in total. Foam padding was used to reduce motion artifacts, and earplugs were provided to lessen noise. All imaging sessions were completed without complications.

#### 2.4.3 Movement scrubbing

**Figure 1:**
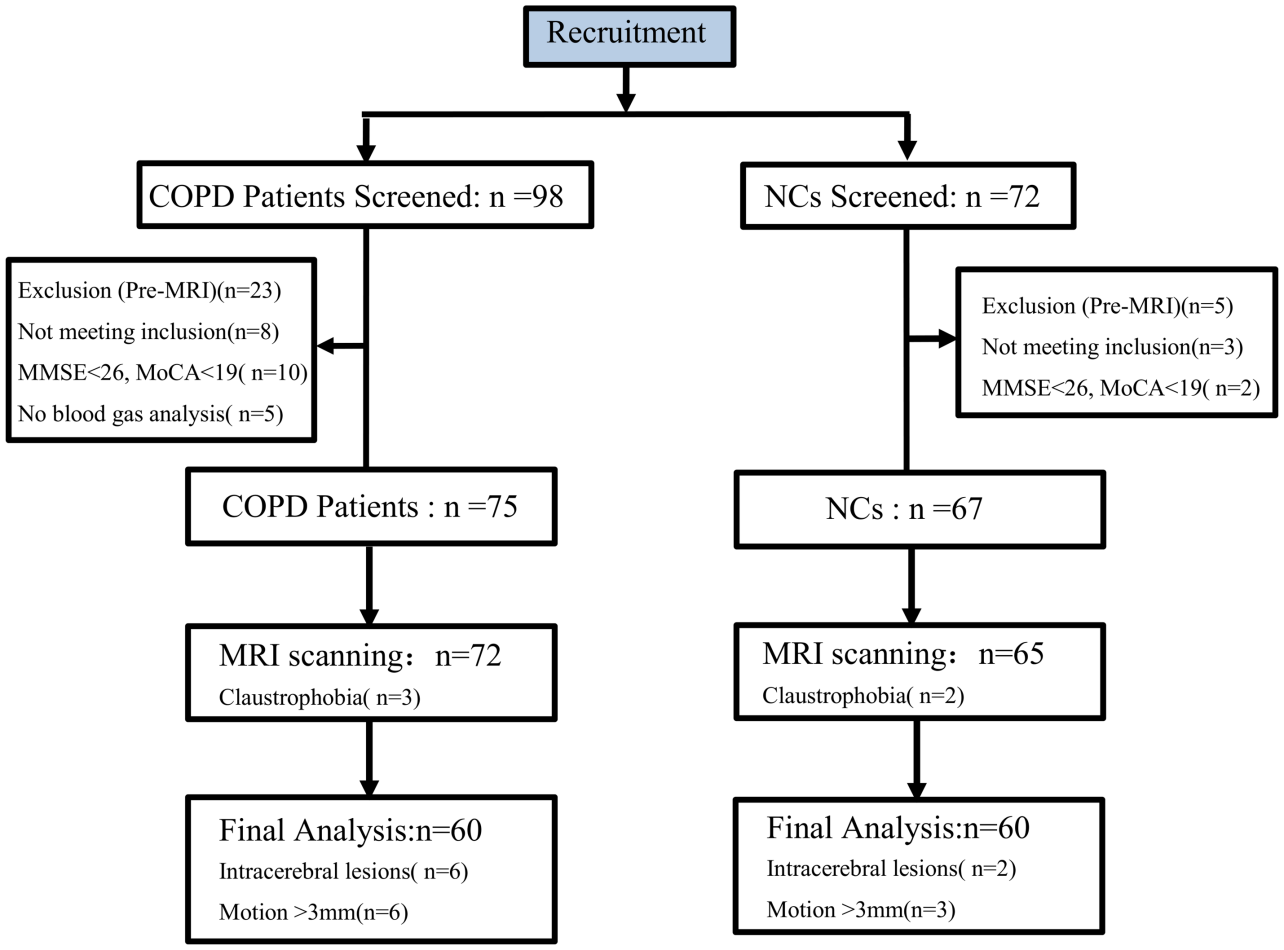
Participant flow diagram. Flowchart illustrating the recruitment and exclusion process; 172 participants were initially assessed; after exclusion for neurological comorbidities and MRI contraindications, 137 underwent scanning. Following strict motion quality control, eight participants were excluded (COPD: *n* = 6; NCs: *n* = 3). Six patients with COPD and two NCs were excluded due to intracerebral lesions. The final cohort consisted of 60 patients with COPD and 60 matched HCs. COPD, Chronic Obstructive Pulmonary Disease; NCs, Normal Controls; MRI, Magnetic Resonance Imaging; MMSE, Mini-Mental State Examination; MoCA, Montreal Cognitive Assessment.

Participants whose maximum head displacement during scanning exceeded 3 mm were either re-scanned or removed from the study (*n* = 6 COPD, *n* = 3 NCs) to maintain data integrity (Figure 1).

### 2.5 Data preprocessing and network reconstruction

Preprocessing and statistical analysis were conducted between January 2023 and March 2024, whereas data were collected between 2012 and 2015. High-resolution T1-weighted structural images were preprocessed using FreeSurfer 7.3^1^ for cortical reconstruction and volumetric segmentation, and FSL 6.0.5^2^ for spatial normalization and quality control. To construct the structural connectome, the brain was parcellated into 90 anatomical regions of interest (ROIs; 45 per hemisphere) using the Automated Anatomical Labeling (AAL) atlas. These ROIs served as network nodes, and WM tracts connecting them were defined as edges.

#### 2.5.1 GM thickness analysis

FreeSurfer version 7.3 was used to analyze T1-weighted images. Cortical surface reconstruction, removal of non-brain tissue, intensity normalization, and motion correction were the four main components of the automated workflow. Vertex-wise cortical thickness maps were produced. We used the Desikan–Killiany atlas to assess regional cortical thickness in order to evaluate our hypotheses. The validity of these morphometric measurements is supported by FreeSurfer’s well-established reliability.

#### 2.5.2 WM tractography

DTI images and their matching structural scans were initially co-registered. The data were standardized to the Montreal Neurological Institute standard space (MNI152) using affine and nonlinear transformations. To increase the signal-to-noise ratio and reduce inter-participant variability, we smoothed the fractional anisotropy (FA) images using an 8-mm full-width at half-maximum Gaussian kernel.

The entire brain was subjected to deterministic tractography using the Diffusion Toolkit, with seeds placed evenly throughout the WM. Streamlines were stopped when the angular deviation was > 35° or the FA value was < 0.15. An automated 90-node anatomical labeling atlas was used to create the connection matrices. To reduce the risk of false positive connections, edges with fewer than four streamlines were removed. For subsequent graph-theoretical analyses, fiber count and mean FA-weighted matrices were generated.

#### 2.5.3 Structural network analysis

Structural connectomes were constructed based on deterministic tractography of DTI data. The brain was parcellated into 90 anatomical ROIs (AAL atlas; 45 per hemisphere). WM fiber counts (or FA values) between ROIs served as edge weights.

To ensure the network comparisons were robust and not biased by a single arbitrary threshold, a sparsity-based thresholding approach was applied. Networks were thresholded across a range of sparsity levels (S = 0.05–0.40) with increments of 0.01. This range ensured that networks remained connected while avoiding non-specific random connections.

For each sparsity level, global and nodal network metrics were calculated using the GRETNA version 2.0 toolbox^3^ to characterize the topological organization of the structural network. Global metrics included global efficiency (E_glob, which describes the overall information transfer efficiency of the network), clustering coefficient (Cp, measuring the connectivity among a node’s neighbors), and shortest path length (Lp, representing the average minimum number of connections required to link any two nodes in the network). Nodal metrics included betweenness centrality (BC, a measure of a node’s connectivity to other nodes, describing its influence on information flow between them), degree centrality (DC, the degree to which a node is directly connected to adjacent nodes, reflecting its information communication capacity), and nodal efficiency (Ne, indicating the efficiency of parallel information transfer at the node level). To ensure physiological interpretability, weighted networks retaining connection strength were constructed. Finally, to summarize topological properties independent of single-threshold selection, the area under the curve (AUC) for each metric across the sparsity range (0.05–0.40) was computed to assess group differences.

#### 2.5.4 Mediation analysis

We conducted a mediation analysis using a path-analytic framework to explore the potential mediating role of the DC of the left supplementary motor area (SMA_L’ DC) in the association between respiratory symptoms (measured using the SGRQ) and cognitive function (measured using the MoCA). The SGRQ total score was designated as the independent variable, the education-adjusted MoCA total score as the dependent variable, and SMA_L’ DC as the mediator. All models were adjusted for age, sex, and education to account for demographic confounding variables.

To calculate indirect effects (a × b) and their bias-corrected and accelerated (BCa) 95% confidence intervals (CIs), we used a non-parametric bootstrapping method with 5,000 resamples. A 95% CI that excluded 0 was considered a significant statistical mediation pattern. Partial mediation was determined if both the direct and indirect effects remained significant, and statistical model consistent with an association pattern if the mediator’s inclusion made the direct effect (c′) non-significant.

### 2.6 Statistical analysis

Group differences in demographic and clinical variables were assessed using IBM SPSS Statistics (v22; IBM Corp., Armonk, NY, United States). Continuous variables are present as mean ± standard deviation. Independent-sample *t*-tests were used for comparisons, and the chi-square test was used for categorical variables. All statistical tests were two-tailed, with *p* < 0.05 considered statistically significant.

Whole-brain voxel-wise analyses of DTI measures (FA and radial diffusivity [RD]) were performed using Statistical Parametric Mapping (SPM). In group comparisons (COPD vs. HCs), age, sex, and education were included as covariates to control for confounding effects. Multiple comparisons were controlled using a voxel-wise threshold of *p* < 0.001 combined with AlphaSim-corrected cluster extent (≥375 voxels), corresponding to a family-wise error (FWE) rate of *p* < 0.05. Statistical maps were overlaid on the MNI152 template for anatomical localization.

To isolate the effect of COPD on brain networks, age, sex, years of education, and smoking status (pack-years) were included as covariates of no interest. Analysis of covariance (ANCOVA) was performed on AUC values of global metrics to compare patients with COPD (*n* = 60) and HCs (*n* = 60). Topological metrics, including Cp, characteristic Lp, E_glob, E_local, and nodal degree, were computed using the GRETNA toolbox. Two-sample *t*-tests were performed on AUC values of nodal metrics across the 90 ROIs. For multiple comparison correction, false discovery rate (FDR) correction was applied for nodal metrics (*q* < 0.05), and Bonferroni correction was applied for global metrics (*p* < 0.05). Additionally, non-parametric permutation testing (5,000 permutations) was conducted for global metrics, yielding results consistent with ANCOVA. Correlations between network measures and clinical parameters (cognitive scores, GOLD stage, blood gas markers, inflammatory indices, and smoking history) were assessed using Pearson or Spearman coefficients, depending on data normality (Shapiro–Wilk test) and homoscedasticity. When assumptions were violated, non-parametric tests were used. The FWE rate was controlled at *α* = 0.05 using Bonferroni correction. Effect sizes (Cohen’s d and partial eta squared [η^2^]) were reported.

A path-analytic framework with 5,000 bootstrap resamples was used to test the statistical mediation model (SGRQ → SMA_L’ DC → MoCA). Bias-corrected and accelerated 95% CIs were calculated for the indirect effect (path a × path b). A significant indirect effect was considered when the indirect effect was significant and the direct effect (route c’) was no longer significant.

### 2.7 Rationale for methodological choices

Surface-based morphometry was selected over volumetric voxel-based approaches due to its greater sensitivity for detecting localized cortical thinning. DTI was used because it accurately depicts the microstructure of WM, including axonal integrity and myelination. By integrating these modalities with graph-theoretical analysis, we obtained a comprehensive view of macroscale structural disconnection. This approach enabled the identification of specific topological substrates underlying domain-specific cognitive deficits. To ensure the robustness and reproducibility of our results, we also employed strict multiple-comparison adjustments and meticulously adjusted for demographic variables.

## 3 Results

### 3.1 Participant flow diagram

A total of 98 patients with COPD and 72 NCs were initially screened. After applying exclusion criteria (neurological disease, MRI contraindications), 75 patients with COPD and 67 controls were eligible for scanning. Following MRI quality control and motion artifact assessment, three patients with COPD and two controls were excluded because of claustrophobia or failed to meet quality standards after re-scanning. Additionally, six patients with COPD and two controls were excluded for intracerebral lesions. The final analysis included 60 patients with COPD-SCV and 60 NCs (Figure 1).

### 3.2 Cognitive and demographic features

Age, sex, BMI, and years of education were all well-matched between the COPD and NC groups (*p* > 0.05). However, patients with COPD had lower health-related quality of life, as indicated by higher SGRQ scores (20.93 ± 12.92), and a substantially heavier smoking history (27.92 ± 9.49 vs. 20.48 ± 12.27 pack-years; *p* = 0.011). Clinically, considerable airflow limitation was observed in patients with COPD in GOLD stages 1–3 (FEV₁: 66.17 ± 18.40% predicted). Every patient was normocapnic (PaCO2: 38.30 ± 5.38 mmHg) and normoxic (PaO2: 80.17 ± 15.44 mmHg). This physiological profile made it possible to separate the consequences of acute gas exchange abnormalities from those of chronic respiratory disease, focusing purely on the neurological effects of COPD.

Global MMSE performance was within the normal range (≥ 27). However, the MoCA revealed mild but significant deficits in the COPD group (25.03 ± 3.13 vs. 27.13 ± 2.32 in NCs; *p* < 0.001). Preliminary screening-level observations suggested potential involvement in name recall, memory, and visuospatial/executive function, although confirmation of these findings requires further detailed neuropsychological assessment (*p* < 0.05). No significant correlation was found between disease duration and cognitive function. This finding suggests that brain impairment in COPD may be more strongly related to underlying pathogenic association patterns, such as systemic inflammation or intermittent hypoxia, than to disease duration. None of the 60 patients with COPD reported subjective cognitive complaints. Therefore, no patients met the full clinical criteria for MCI, supporting the “subclinical” nature of this cohort (COPD-SCV).

### 3.3 Multimodal neuroimaging signatures of COPD-related brain alterations

#### 3.3.1 Regional GM atrophy and WM disruption

**Figure 2:**
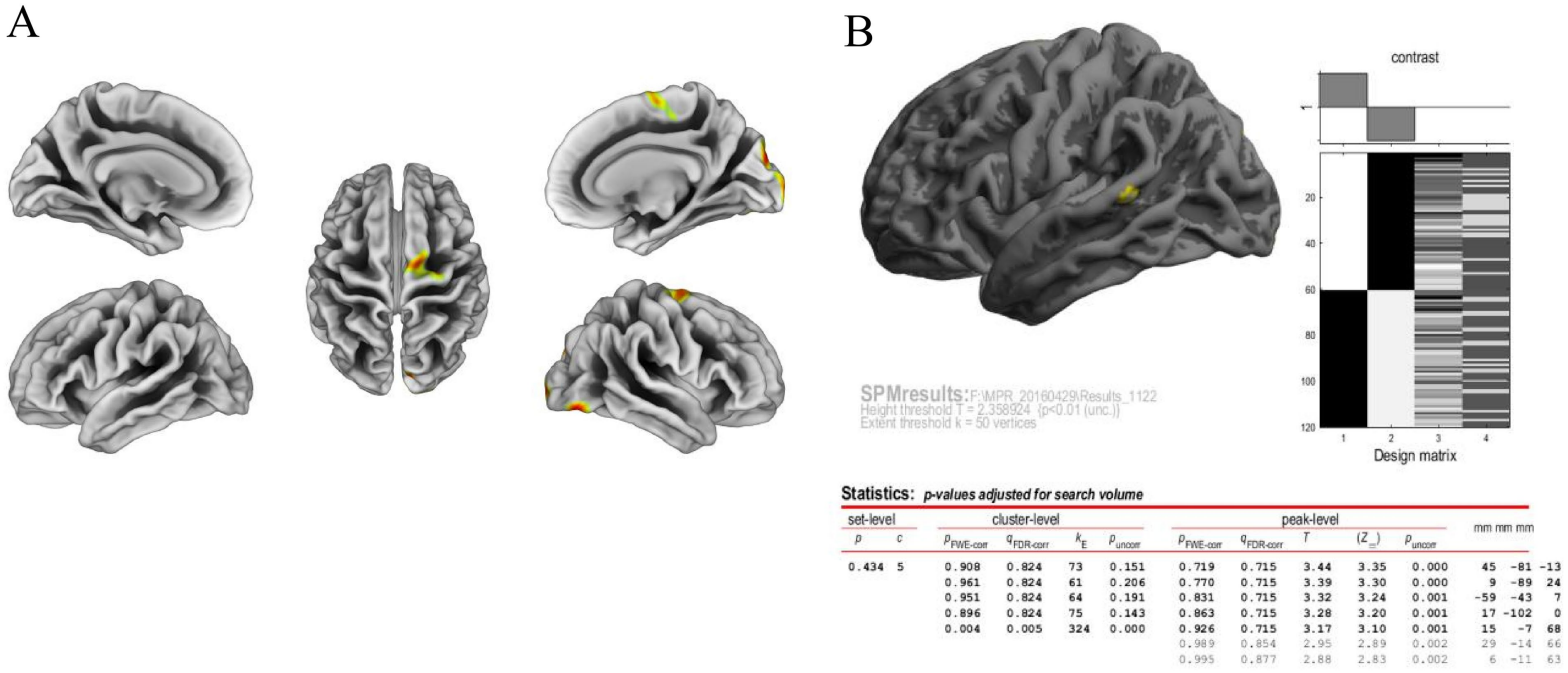
Cortical thinning in COPD: voxel-wise mapping of important areas and related brain structures. There are notable variations in cortical thickness between patients with COPD and NCs. **(A)** Brain areas showing significant cortical thinning in the COPD group compared to that in NCs were identified using voxel-wise statistical parametric mapping (*p* < 0.01). Clusters where cortical thickness is markedly lower in patients with COPD are represented by the color-coded areas. **(B)** 3D representation of the important clusters identified in **(A)** and a comprehensive statistical summary of the five major brain areas that correlate with the peak coordinates inside these clusters. The affected areas are IOG.R, cuneus.R, STG.L, calcarine.R, and SFG.R. COPD, chronic obstructive pulmonary disease; IOG.R, right inferior occipital gyrus; cuneus.R, right cuneus; STG.L, left superior temporal gyrus; calcarine.R, right calcarine; SFG.R, right superior frontal gyrus.

In individuals with COPD, significant cortical thinning was observed in distinct brain regions, including the right occipital lobe (inferior occipital gyrus, cuneus, and calcarine), right SMA, superior temporal gyrus (STG), and right superior frontal gyrus (SFG; Figure 2; Table 1). The visual and sensory-motor domains were mainly related to these alterations. Remarkably, there was no cortical thickening in the brain, suggesting a pattern of progressive, yet potentially modifiable, brain changes that align with the concept of accelerated brain aging in COPD (Yu et al., 2025).

**Table 1:** Cortical thickness alterations in COPD relative to NCs.

Cluster	Brain regions	MNI coordinates			Peak *t*-value	Number of voxels	*p*-value
		*x*	*y*	*z*			
Cluster 1	Inferior occipital_R	45	−81	−13	3.44	3,543	<0.01
Cluster 2	Cuneus_R	9	−89	24	3.39	184	<0.01
Cluster 3	Middle temporal_L	−59	−43	7	3.32	220	<0.01
Cluster 4	Calcarine_R	17	−102	0	3.28	298	<0.01
Cluster 5	Supplementary Motor Area_R	15	−7	68	3.17	207	<0.01

The threshold set as *p* < 0.01, Cluster number>50, false discovery rate corrected. COPD, Chronic Obstructive Pulmonary Disease; NCs, Normal Controls; MNI, Montreal Neurological Institute; L, left; R, right.

**Figure 3:**
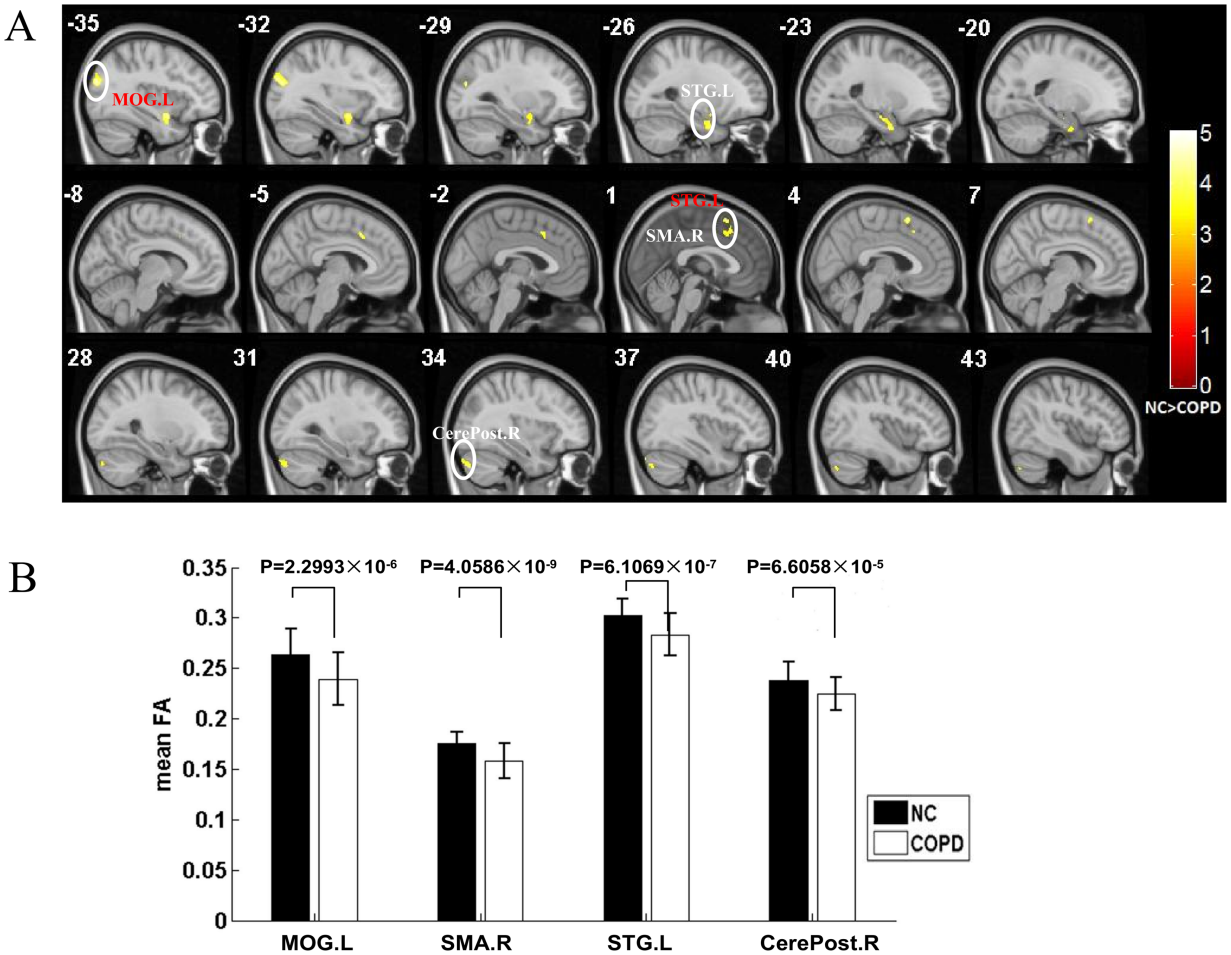
Reduced FA in COPD WM integrity: a voxel-base mapping and regional quantitative analysis. Compared to NCs, patients with COPD have a different WM microstructure as determined by FA. **(A)** Voxel-wise FA reductions. Compared to NCs, patients with COPD have considerably lower FA levels (*p* < 0.001). Significant clusters of reduced FA are represented by the color-coded regions (yellow), found in the MOG.L, SMA.R, STG.L, and CerePost.R. **(B)** Mean FA values. Patients with COPD (white bars) show significantly lower FA levels than those of NCs (black bars) in all four designated regions: MOG.L (*p* = 0.032), SMA.R (*p* = 0.018), STG.L (*p* = 0.041), and CerePost.R (*p* = 0.025). COPD, Chronic Obstructive Pulmonary Disease; FA, Fractional Anisotropy; WM, White Matter; NCs, Normal Controls; MOG.L, left middle occipital gyrus; SMA.R, right supplementary motor area; STG.L, left superior temporal gyrus; CerePost.R, right cerebellum posterior lobe.

**Figure 4:**
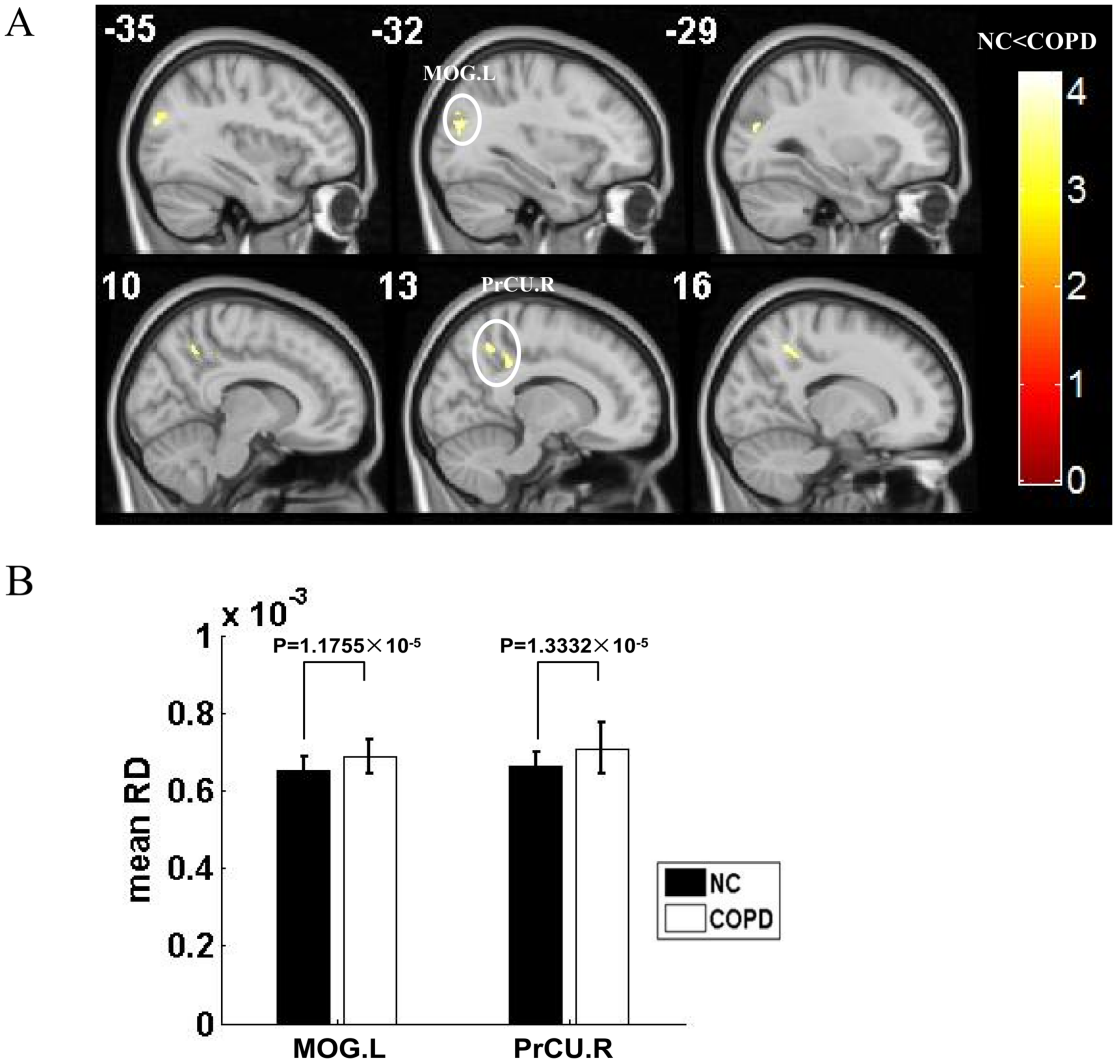
Increased RD in COPD WM: a voxel-base mapping and regional quantitative analysis. Changes in WM RD between patients with COPD and NCs. **(A)** Voxel-based changes in RD. Warm colors (red/yellow) indicate clusters where patients with COPD show considerably higher RD (*p* < 0.001). The MOG.L and the PrCU.R are where the most noticeable clusters are located. **(B)** Bar charts showing average RD values. Patients with COPD (white bars) show significantly higher RD values than those of NCs (black bars) in both the MOG.L (*p* < 0.05) and PreCU.R (*p* < 0.05). COPD, Chronic Obstructive Pulmonary Disease; RD, Radial Diffusivity; WM, White Matter; NCs, Normal Controls; MOG.L, left middle occipital gyrus; PrCU.R, right precuneus.

Widespread WM microstructural damage coincided with these cortical alterations. DTI analysis revealed significantly lower FA in the left middle occipital gyrus, right SMA, left STG, and right posterior cerebellar lobe. At the same time, RD was higher in left middle occipital gyrus and right precuneus (Figures 3, 4; Tables 2, 3). Demyelination and axonal degradation were indicated by the co-localization of lower FA and higher RD, especially in long-range association tracts connecting occipital, temporal, and motor regions.

**Table 2:** Decreased FA value of white matter for the COPD group compared with NCs group (*p* < 0.001, cluster size >397 voxels, AlphaSim corrected).

Brain regions	Brodmann area (BA)	MNI coordinates			Cluster size (mm^3^)	*T* value	*Z* value
		*x*	*y*	*z*			
MOG_L	19	−34	83	23	895	4.87	4.64
SMA_R	6/8/32	3	5	59	761	4.54	4.19
		−5	16	39		3.96	3.83
		2	22	45		3.85	3.72
STG_L		−34	0	−17	1,418	4.45	4.26
		−24	−2	−31		4.06	3.92
		−22	−11	−21		3.76	3.64
CerePost_R		33	−81	−36	585	3.98	3.84
		41	−74	−41		3.64	3.54
		36	−86	−20		3.50	3.40
						

FA, Fractional Anisotropy; COPD, Chronic Obstructive Pulmonary Disease; NCs, Normal Controls; MNI, Montreal Neurological Institute; MOG.L, left middle occipital gyrus; SMA.R, right supplementary motor area; STG.L, left superior temporal gyrus; CerePost.R, right cerebellum posterior lobe.

**Table 3:** Decreased RD value of white matter for the COPD group compared with that of the NCs group (*P* < 0.001, cluster size >397 voxels, AlphaSim corrected).

Brain regions	Brodmann area (BA)	MNI coordinates			Cluster size (mm^3^)	*T* value	*Z* value
		*x*	*y*	*z*			
MOG_L	19	−34	83	23	578	4.19	4.03
		−30	−83	19		4.07	3.93
PrCU_R	7	15	−53	48	582	4.12	3.97
		13	−39	36		3.92	3.79
						

RD, Radial Diffusivity; COPD, Chronic Obstructive Pulmonary Disease; NCs, Normal Controls; MNI, Montreal Neurological Institute; MOG.L, left middle occipital gyrus; PrCU.R, right precuneus.

#### 3.3.2 Topological inefficiency of the structural connectome

**Figure 5:**
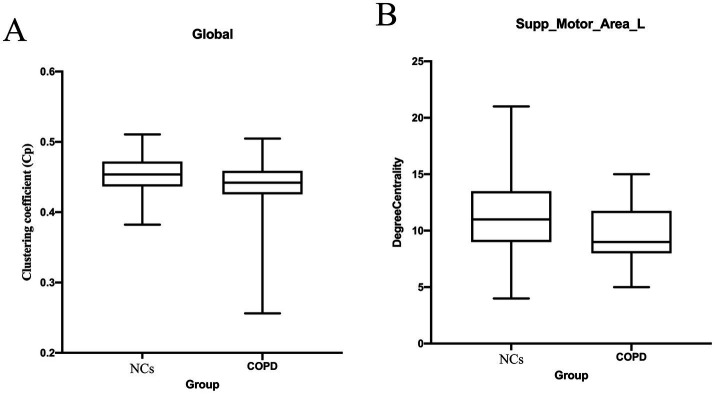
Global integration and nodal centrality are reduced in COPD due to disruptions in the topological architecture of structural networks. Alterations in the topological characteristics of structural brain networks in patients with COPD compared with those in NCs. **(A)** Global network property: Cp. The global clustering coefficient distribution for each group is displayed in the box plot. **(B)** Nodal network property: Supp_Motor_Area_L′ DC. When compared to NCs, the COPD group shows considerably lower DC in Supp_Motor_Area_L′ DC, which is shown in the box plot (*p* < 0.05). COPD, Chronic Obstructive Pulmonary Disease; NCs, Normal Controls; Cp, Clustering Coefficient; Supp_Motor_Area_L, Left supplementary motor area; DC, Degree Centrality.

The brain networks of patients with COPD showed extensive topological remodeling, according to graph-theoretical analysis. Patients with COPD showed a marked decrease in global Cp and network efficiency, indicating a departure from an ideal “small-world” architecture and lower ability to integrate information (Figure 5; Table 4). To further validate the robustness of our findings and control for potential confounding by smoking status, we repeated the mediation analysis with pack-years included as an additional covariate. The results remained largely consistent with those of the initial analysis (Table 4). DC was considerably decreased at the nodal level in the insular cortex and SMA_L, which are important hubs for interoceptive processing and respiratory regulation. Together, these findings suggest that COPD disrupts the delicate balance between functional segregation and integration, which lowers the brain’s ability to efficiently process local and global information.

**Table 4:** Comparison of graph-theoretical metrics of structural brain networks between COPD patients and NCs.

Metric	COPD (*n* = 60)	NCs (*n* = 60)	*P* value	P^*^ value
Global network				
Clustering coefficient, Cp	0.44 ± 0.04	0.45 ± 0.03	0.007	0.010
Network efficiency	0.65 ± 0.06	0.67 ± 0.04	0.018	0.210
Nodal				
Degree centrality				
Supplementary motor area_L	9.20 ± 3.14	11.07 ± 2.75	0.000748(FDR-corrected *P* < 0.05)	0.0017 (FDR-corrected *P* < 0.05)
Insula_L	9.60 ± 2.61	11.47 ± 3.22	0.000683(FDR-corrected *P* < 0.05)	0.00087(FDR-corrected *P* < 0.05)

Metrics represent the area under the curve across the sparsity range (0.10–0.40). Group comparisons were performed using the analysis of covariance. *p*-values were adjusted for age, sex, and years of education; p*, further adjusted for smoking status (pack-years). Nodal degree centrality was FDR-corrected for multiple comparisons (*q* < 0.05). COPD, Chronic Obstructive Pulmonary Disease; NCs, Normal Controls; Cp, Clustering Coefficient; AUC, Area Under the Curve; FDR, False Discovery Rate; ANCOVA, Analysis of Covariance.

#### 3.3.3 Structure–function-symptom coupling

To link changes in neuroanatomy with clinical symptoms, we examined the relationship between imaging metrics and functional outcomes. At the regional level, the right cuneus thickness was positively associated with language and verbal fluency, whereas the left STG thickness was associated with attention and visuospatial/executive skills. Lower MoCA scores were independently associated with lower E_glob at the network level (*β* = 0.340, *p* < 0.01).

Multivariate regression analysis utilizing morphometric, microstructural, and topological features accounted for a significant portion of the variance in cognitive function. Right cuneus thickness and E_glob emerged as the strongest independent predictors of MoCA scores. Furthermore, a distinct pattern that predicted the intensity of respiratory symptoms (SGRQ scores) was identified, characterized by lower E_glob and higher RD in the precuneus. These findings indicate a shared pathophysiological substrate that concurrently compromises respiratory function and cerebral microstructural integrity, potentially involving hypoxic–ischemic damage or neuroinflammation.

#### 3.3.4 Mediation analysis: SMA connectivity as a neural bridge

**Figure 6:**
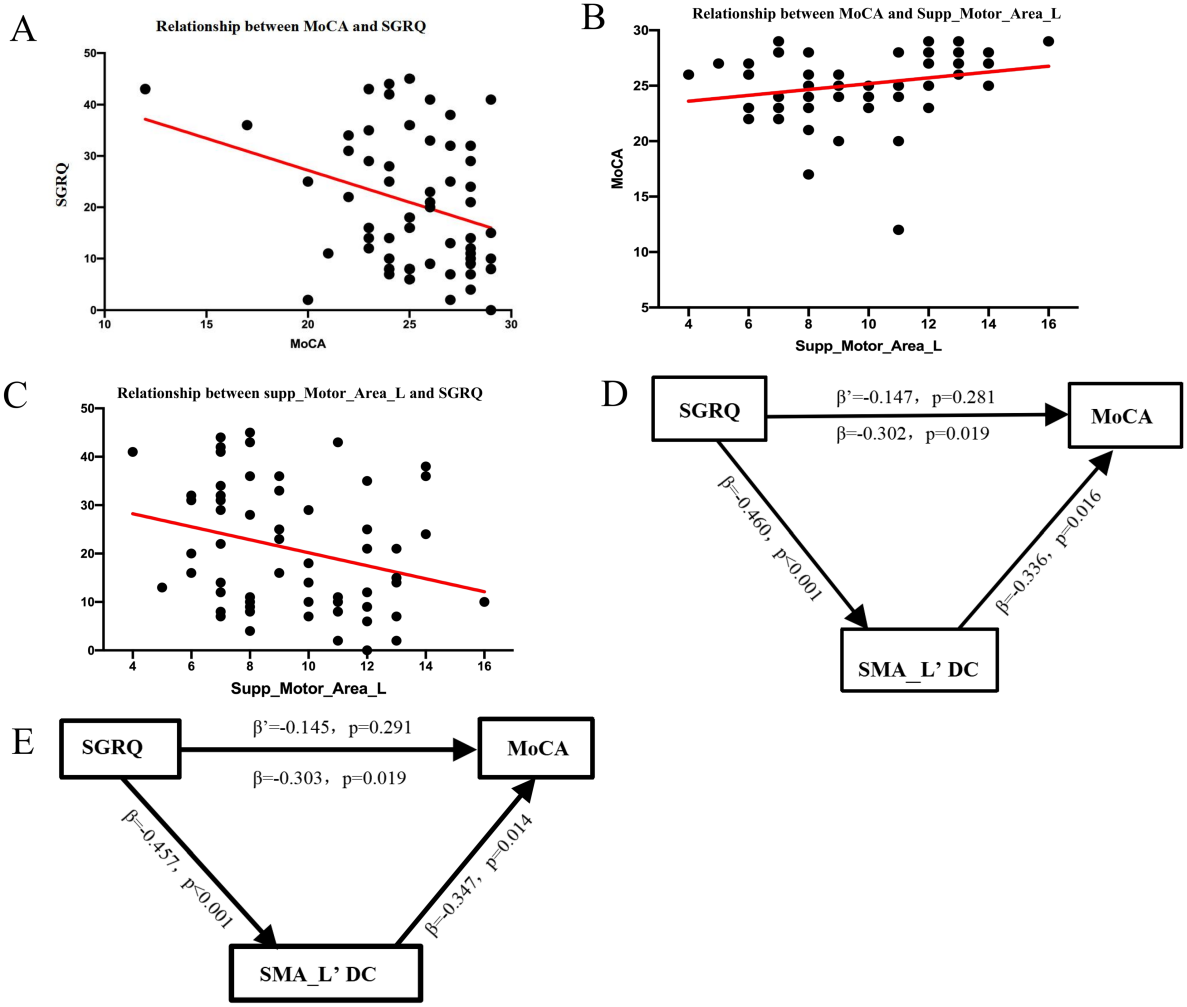
The Supp_Motor_Area_L as a neural hub: DC the link between COPD respiratory symptoms (SGRQ) and global cognitive impairment (MoCA). The relationship between SGRQ and MoCA is mediated by structural network hub integrity. **(A)** Fitted regression line scatter plot reveals a significant negative association between the total MoCA score and total SGRQ score for all patients (*r* = −0.52, *p* < 0.001). **(B)** Scatter plot showing that MoCA scores and the SMA.L′ DC have a strong positive correlation (*r* = 0.45, *p* = 0.003). **(C)** Scatter plot indicating that the SMA.L′ DC and SGRQ scores have a strong negative correlation (*r* = −0.41, *p* = 0.007). **(D)** Mediation analysis, with the path diagram showing quantifications of the proposed mediation model. The coefficients for the standardized path are displayed. When the mediator (SMA.L′ DC) was added to the model, the direct effect of SGRQ on MoCA (path c′) decreased and became non-significant (*β* = −0.28, *p* = 0.08). Importantly, there was a substantial indirect effect through SMA.L′ DC (*β* = −0.24, 95% confidence interval: [−0.42, −0.10]). **(E)** Results of mediation analysis after adding smoking status as a covariate; results are consistent with the initial results without including smoking status as a covariate. This cross-sectional model represents a statistical association rather than confirmed causality. COPD, Chronic Obstructive Pulmonary Disease; SGRQ, St. George’s Respiratory Questionnaire (SGRQ); MoCA, Montreal Cognitive Assessment; SMA_L, Left supplementary motor area; DC, Degree Centrality.

We hypothesized that structural network integrity is associated with the relationship between respiratory burden and cognitive function. To explore this hypothesis, mediation analysis was conducted focusing on the SMA_L, a key hub exhibiting nodal atrophy and low centrality. The results indicated a significant association pattern consistent with mediation (Figure 6).

The initial mediation analysis demonstrated a significant statistical association between respiratory symptoms (SGRQ) on cognitive performance (MoCA) (Path c: *β* = −0.302, *p* = 0.019), consistent with a statistical mediation model. Higher symptom burden was associated with lower DC in the SMA_L (Path a: *β* = −0.460, *p* < 0.001), which in turn was associated with poorer cognitive performance (path b: *β* = 0.336, *p* = 0.016). After accounting for this mediator, the direct effect of SGRQ on MoCA was no longer significant (path c’: *β* = −0.147, *p* = 0.281), suggesting a statistically significant mediation effect by SMA_L’DC.

To further validate robustness and control for smoking, the mediation analysis was repeated with smoking pack-years as a covariate. The results remained consistent. Respiratory symptoms continued to show a significant total effect on cognitive performance (path c: *β* = −0.303, p = 0.019). Similarly, higher symptom burden was associated with lower SMA_L’DC (path a: *β* = −0.457, *p* < 0.001), which predicted poorer cognitive performance (path b: *β* = 0.347, *p* = 0.014). The direct association remained non-significant (path c’: *β* = −0.145, *p* = 0.291).

These findings suggest that respiratory dysfunction is indirectly associated with cognitive performance through disruption of structural connectivity in the SMA_L, a key node in the respiratory motor network. Importantly, adjustment for smoking did not alter the significance or direction of results, supporting the robustness of the statistical mediation model.

## 4 Discussion

The aim of this study was to define the neuroanatomical foundations of COPD-SCV in normoxic patients, extending beyond the traditional research paradigm that focuses on substantial cognitive impairment (MCI/dementia) and hypoxia. Our primary findings reveal a distinct pattern of brain structural deterioration in patients with COPD-SCV, including regional GM atrophy, WM microstructural breakdown, and topographical inefficiency. These changes were associated with specific deficits in visuospatial and executive domains rather than global cognitive impairment, even in the absence of major abnormalities in gas exchange and global cognitive decline (as indicated by preserved MMSE scores). Crucially, the association between cognitive impairment and respiratory symptom burden was statistically mediated by the SMA_L’DC. These results identify a statistically supported association pattern consistent with a refined “lung-brain axis” model (Yu et al., 2025), suggesting that the neurological effects of COPD may be driven by targeted disruption of higher-order cortical networks linked to respiratory symptom burden, rather than hypoxia alone.

### 4.1 Refining the concept of COPD-SCV: a pre-MCI phenotype

This study introduces COPD-SCV as an operational study label to explore a distinct phenotype preceding MCI (pre-MCI). Unlike the retrospective label of “pre-MCI,” COPD-SCV is defined prospectively in this study by preserved global cognition (MMSE ≥ 27), intact ADL, and the absence of subjective cognitive complaints, while exhibiting subtle cognitive deviations detectable by sensitive instruments such as the MoCA. However, external validation is needed to establish COPD-SCV as a clearly defined clinical entity. This definition captures a “silent” window of neurodegeneration in which structural atrophy outpaces functional impairment. The relative preservation of hippocampal volume in our cohort—contrasting with studies linking hippocampal atrophy to advanced COPD (Zhang J. et al., 2013; Esser et al., 2015) supports a temporal hierarchy of neuropathology: frontoparietal and occipital integration hubs deteriorate first, whereas medial temporal lobe atrophy may be a downstream event leading to overt dementia. This operational definition is critical: by requiring preserved MMSE (≥ 27) and no subjective complaints, we deliberately excluded the clinical diagnosis of MCI. This allows us to capture a unique “at-risk” state in which neurodegeneration is active but has not yet manifested as clinical symptoms, distinguishing COPD-SCV from HCs and patients with MCI.

### 4.2 Multimodal neuroimaging signatures: beyond hypoxia

Regional GM atrophy, WM deterioration, and topological inefficiency were observed in patients with COPD-SCV. Although consistent with previous research describing widespread atrophy and WM integrity loss in COPD (Li and Fei, 2013; Zhang J. et al., 2013; Wang et al., 2017), our findings diverge from studies focusing on patients with overt cognitive impairment or severe hypoxemia, which typically report diffuse global atrophy (Zhang J. et al., 2013; Esser et al., 2015). Instead, our cohort—characterized by COPD-SCV (defined by preserved global cognition [MMSE ≥ 27], functional independence, and subtle MoCA deficits), the absence of hypoxemia, and mild-to-moderate airflow limitation (GOLD 1–3)—exhibited a more subtle, spatially restricted pattern of damage. Multimodal neuroimaging identified that GM atrophy predominantly involved the right inferior occipital gyrus (IOG), cuneus, and SFG. This challenges the “hypoxia-centric” model, suggesting that systemic inflammation and oxidative stress—rather than hypoxemia—drive selective vulnerability in metabolically active association cortices.

This spatial specificity offers a distinct perspective on the early neuropathology of COPD and aligns with the concept of accelerated brain aging. Recent consensus on brain aging biomarkers proposes that systemic inflammatory conditions, such as COPD, may accelerate age-related neurodegenerative trajectories rather than follow a singular, irreversible pathway (Aging Biomarker et al., 2023). We propose that metabolically active association cortices—particularly those involved in visuospatial processing and executive control—exhibit selective vulnerability to systemic inflammation and oxidative stress, even in the absence of hypoxia. This posterior-dominant pattern suggests a pathophysiological trajectory distinct from normal aging or Alzheimer’s disease. Furthermore, the relative preservation of hippocampal volume in our subclinical population—contrasting with research highlighting hippocampal atrophy as a marker of deterioration in advanced COPD (Zhang J. et al., 2013; Esser et al., 2015)—supports a temporal hierarchy of neuropathology. We hypothesize that deterioration of frontoparietal and occipital integration hubs marks the incipient subclinical stage (COPD-SCV), whereas medial temporal lobe atrophy may be a downstream event leading to overt dementia.

Our DTI results provide a granular view of WM microstructure, revealing lower FA and higher RD in tracts connecting these regions (e.g., left middle occipital gyrus, right SMA, left STG), indicating demyelination and axonal degradation in tracts connecting sensorimotor and visual processing regions. Complementing this, structural network analysis fills a key gap in COPD connectomics. We employed a rigorous graph-theoretical approach using the AAL-90 atlas. By calculating the AUC across a sparsity range (0.05–0.4), we ensured robustness against threshold selection bias. FDR correction was applied to nodal metrics to control for multiple comparisons. Graph-theoretical analysis revealed systemic topological disruption, including lower nodal centrality and lower global efficiency. These findings indicate a departure from the optimal “small-world” architecture, suggesting a shift in which the brain prioritizes local processing over global integration—a hallmark of accelerated network aging. Notably, these topological metrics may be more sensitive to early changes than volumetric measures alone, as network disintegration may precede functional impairment in memory-related regions.

### 4.3 Structure–function coupling: dysconnectivity and altered network topology

A strong association exists between cognitive decline and structural alterations in COPD-SCV. Preliminary observations from screening-level assessments suggest a potential correlation between visuospatial task performance and atrophy in the cuneus and occipital gyri, regions anatomically linked to the dorsal visual stream. Similarly, screening-level findings hint at possible associations between executive and semantic retrieval task performance and thinning of the right SFG and STG. However, these findings have to be validated using detailed neuropsychological evaluation. Similarly, decision-making and planning impairments co-occur with atrophy in the right SFG, a critical region associated with the executive control network, while naming difficulties align with atrophy in the left STG.

Beyond isolated regions, our network analysis extends these findings to the connectome. The DC of the insular cortex, a key hub for interoception and salience, was lower. Given the insula’s role in integrating bodily states with cognitive control and processing dyspnea, its disruption may represent a critical interface through which respiratory distress manifests as cognitive dysfunction. This observation aligns with the concept of connectome vulnerability, wherein hub regions (e.g., SMA, insula) exhibit reduced DC and global efficiency, leading to cognitive decline. This is supported by recent graph-theoretical analyses that link hub disruption to reduced network efficiency in neurodegenerative conditions (Fornito and Bullmore, 2012). In addition, this observation aligns with the “dysconnectivity hypothesis” of cognitive aging and neurodegeneration (Filippi et al., 2013), which posits that cognitive deterioration arises from disrupted structural and functional communication pathways, particularly affecting high-cost, long-range connections managed by critical network hubs (Stam, 2014). The ongoing burden of respiratory symptoms in COPD may act as a chronic stressor, accelerating the breakdown of these high-demand hubs and impairing information transfer across the frontoparietal control network.

### 4.4 A statistically supported lung-brain pathway (mediation analysis)

This pattern of structural-functional coupling offers a statistically supported association consistent with a lung-brain pathway. Our preliminary findings, based on screening-level cognitive assessments, suggest that the subjective burden of respiratory symptoms (SGRQ) aligned with a behavioral proxy for allostatic load on central executive centers. This could explain why patients with higher symptom burdens show lower cognitive performance on screening tests, though detailed neuropsychological profiling is needed for confirmation, independent of disease duration or FEV_1_. Given the cross-sectional design of the current study, mediation pattern of SMA connectivity has to be interpreted cautiously. Rather than proving a definitive causal mechanism in which symptoms directly damage the brain, our findings suggest a statistical mediation model in which structural network integrity accounts for the link between respiratory burden and cognition. This association is consistent with the hypothesis that chronic dyspnea may be associated with metabolic demands on supramodal hubs such as the SMA, but it does not establish temporal precedence. Consequently, although the SMA acts as a statistical bridge in this associative pathway, longitudinal validation is required to confirm whether reducing symptom burden can preserve network integrity and cognitive function.

The significant association between SMA_L’DC, respiratory symptom load (SGRQ), and cognitive performance (MoCA) is a key finding of this study, which indicates a correlational consistent with the lung–brain axis hypothesis. However, our findings do not provide evidence for a definitive biological mechanism owing to the cross-sectional study design (Yu et al., 2025; Han et al., 2025). Specifically, our data suggest that SMA_L connectivity may serve as a critical nodal point linking respiratory burden to cognitive outcomes, consistent with—but not proving—a lung-brain pathway. The SMA is recognized as a supramodal hub for cognitive control, task switching, and the execution of complex behavioral sequences, extending beyond its traditional role in motor planning. Recent connectome studies confirm the role of the SMA as an integrative hub linking sensorimotor processing with higher-order executive control networks (Bala et al., 2025), making it a plausible candidate for mediating the cognitive impact of chronic bodily distress. Although prior literature has established a link between SGRQ scores and cognitive deterioration (von Siemens et al., 2019), our multimodal approach identifies the SMA_L as a specific anatomical locus where this clinical-cognitive relationship converges. Given its dense vascularization, high mitochondrial density, and position as a connector between the limbic system and the frontoparietal control network, we hypothesize that the SMA may be particularly susceptible to the metabolic burden associated with chronic dyspnea. This is consistent with the framework of allostatic load, which posits that the cumulative physiological burden of chronic stressors—such as persistent dyspnea (Yu et al., 2016)—can lead to structural and functional brain changes (Palix et al., 2025). The constant metabolic demand required to manage respiratory symptoms and interoceptive distress may contribute to a high allostatic load, preferentially affecting highly connected and metabolically active brain hubs such as the SMA (Mickle et al., 2023; Booth et al., 2015). This chronic over-recruitment could lead to excitotoxicity, reduced neurotrophic support, and eventual structural degradation, providing a plausible biological mechanism for the observed mediation. Consequently, the SMA exhibits statistical associations that reflect how the allostatic burden of respiratory symptoms is related to structural network alterations. Furthermore, this pattern offers a potential framework for understanding the relative contributions of hypoxia versus symptom burden in the literature. Although hypoxia is a well-known neurotoxin, our findings suggest that in non-hypoxemic patients, the perception and burden of respiratory symptoms (as measured by the SGRQ) acts as an objective indicator of the homeostatic stress placed on central executive centers. This subjective burden predicts brain health more robustly than physiological measures alone, suggesting that non-hypoxic pathways may play a significant role in early neurodegeneration. Clinically, identifying SMA_L’DC as a statistical mediator shifts the perspective on risk stratification. In our cohort, traditional predictors of disease progression, such as disease duration and GOLD stage, did not significantly predict cognitive status. In contrast, structural alterations in the SMA were strongly associated with symptom burden (SGRQ). This association suggests that clinical management might benefit from targeting symptom load reduction to preserve the structural integrity of the SMA and potentially safeguard cognitive function in the aging COPD brain, rather than focusing solely on pulmonary function metrics. However, it is crucial to emphasize that these clinical implications are derived from cross-sectional associations and require validation in longitudinal intervention studies to establish causality.

### 4.5 Strengths, limitations and future directions

#### 4.5.1 Strengths

This study has several notable strengths. First, it focuses on a clinically meaningful and relatively underexplored stage of COPD-SCV, namely patients with preserved MMSE but abnormal or subtly lower cognition as assessed by the MoCA. By studying normoxic patients with COPD-SCV, we identified a critical window with emerging neural alterations, separating neural pathology from oxygen deprivation and challenging the “hypoxia-centric” theory. Second, the multimodal MRI framework is a major strength and provides a broader systems-level perspective than a single-modality study. Third, the matched-control design, motion control, and use of multiple comparison procedures indicate efforts to improve analytic rigor. Finally, the convergence of cognitive, cortical, WM, and network-level findings increases the biological plausibility of the overall pattern reported.

#### 4.5.2 Limitations

##### 4.5.2.1 Residual confounding by smoking

Although we adjusted for smoking pack-years in our models, we acknowledge that this metric is an imperfect surrogate for cumulative tobacco exposure. Pack-years do not fully capture critical variables such as smoking intensity (cigarettes/day), duration of exposure, or time since cessation, all of which may independently associate with brain atrophy and cognitive reserve. Furthermore, tobacco smoke exerts complex vascular and pro-inflammatory effects that may accompany irreversible microvascular damage, potentially confounding the structural changes attributed to COPD alone. Although the persistence of the significant mediation pattern after adjustment suggests that COPD-specific pathophysiology (e.g., hypoxia, systemic inflammation) plays a distinct role, we cannot entirely disentangle the effects of COPD from the neurotoxic legacy of smoking. Therefore, the observed reduction in SMA connectivity should be interpreted as a convergent pathway relate to COPD and smoking. Future longitudinal studies with non-smoker COPD cohorts (e.g., biomass exposure) are essential to isolate disease-specific associations and confirm that SMA atrophy is not merely a biomarker of tobacco use.

##### 4.5.2.2 Physiological data in controls

The absence of ABG/spirometry in HCs is a limitation. This was an ethical decision to avoid invasive procedures in healthy volunteers. Instead, we relied on rigorous health questionnaires and exclusion criteria to ensure that HCs were free of respiratory symptoms. Although this introduces uncertainty regarding “subclinical” lung abnormalities in HCs, the normoxic status of the COPD cohort (PaO₂ > 60 mmHg) and significant SGRQ differences suggest that the observed brain changes are driven by symptom burden rather than undetected hypoxia in controls. Nevertheless, this limits direct correlation of brain structure with pulmonary function across the full health spectrum.

##### 4.5.2.3 Cross-sectional design and causal inference

The most critical limitation is the cross-sectional nature of the mediation analysis. Although the statistical model (SGRQ → SMA_L’DC → MoCA) fits the data well and is consistent with a lung-brain pathway, it does not establish temporal precedence. We cannot definitively rule out reverse causality (e.g., cognitive decline leading to altered perception of dyspnea) or unmeasured third variables (e.g., systemic inflammation affecting lung and brain simultaneously). Therefore, the neural correlate explanation of the “lung-brain axis” presented in this study should be regarded as hypothesis-generating. Longitudinal studies are required to validate whether changes in SMA connectivity precede cognitive decline and whether reducing respiratory symptom burden can preserve network integrity.

##### 4.5.2.4 Exclude neurodegenerative disorders

Another limitation of our study is the reliance solely on clinical history to exclude neurodegenerative disorders. Given the age of our cohort, some participants may have been in the preclinical or prodromal stages of neurodegenerative diseases, such as Alzheimer’s disease or Parkinson’s disease, without overt clinical symptoms. This could potentially confound our findings, as these conditions can also affect brain structure and cognitive function. Future studies should incorporate biomarker confirmation, such as CSF analysis for amyloid and tau proteins or PET imaging, to more definitively exclude these conditions and ensure the specificity of our findings to COPD-related brain changes.

Additionally, some limitations must be acknowledged. The lack of direct molecular validation limits the ability to link structural atrophy to cytokine-mediated pathways. Reliance on the MoCA does not eliminate the need for comprehensive neuropsychological assessments. Data collected between 2012 and 2015 should be interpreted with caution regarding technological generalizability; however, the concept of network disintegration remains valid.

#### 4.5.3 Future directions

To definitively establish causality and move beyond associative cross-sectional findings, future research on neurodegeneration in COPD must prioritize longitudinal cohort studies. Specifically, tracking cognitively normal patients with COPD-SCV over extended periods is essential to determine whether alterations in SMA connectivity precede cognitive decline or merely co-occur with it. Such designs will clarify whether SMA_L connectivity may be a predictive biomarker for the conversion from COPD-SCV to MCI, addressing the temporal ambiguity inherent in current cross-sectional models. Furthermore, to dissect the metabolic-inflammatory axis underlying these network disruptions, future studies should integrate multimodal neuroimaging with molecular biomarkers (e.g., cytokines, neurofilament light chain). Advanced imaging techniques—including high-resolution structural connectivity, molecular PET, arterial spin labeling, and functional MRI—will help elucidate the etiology of network failure. This multimodal approach is critical for verifying whether hypoperfusion and neuroinflammation are primary drivers of structural degradation in the SMA and associated hubs. Ultimately, these efforts should aim to identify the most sensitive MRI biomarkers for predicting the transition from normal cognition to MCI. By capturing dynamic imaging trajectories before and after cognitive decline, hypotheses can be generated regarding which network metrics are most responsive to therapeutic intervention, paving the way for targeted strategies to preserve cognitive function in the aging COPD brain. Future research should also focus on biomarker confirmation to exclude preclinical or prodromal neurodegenerative disorders more definitively. This will enhance the specificity of findings and ensure that observed brain changes are attributable to COPD rather than other age-related neurodegenerative processes.

### 4.6 Clinical implications

Despite these limitations, our findings have clinical relevance. SGRQ scores—a patient-reported outcome—predicted brain network changes better than FEV_1_ or GOLD stage. This suggests that symptom burden is a more sensitive indicator of cerebral risk than spirometry alone. Clinically, aggressive management of dyspnea (e.g., pulmonary rehabilitation, anxiolytics) may not only improve quality of life but also preserve structural network integrity in the SMA, potentially delaying cognitive decline.

## 5 Conclusion

This multimodal MRI study identifies a distinct neuroanatomical phenotype of COPD-SCV, characterized by occipito-frontal atrophy, WM microstructural disruption, and topological inefficiency, independent of acute hypoxemia. We identified an association in which structural connectivity of the SMA_L aligned with a key nodal interface linking respiratory symptom burden to cognitive impairment. These findings align with the lung-brain axis hypothesis, suggesting that metabolic strain from chronic dyspnea targets high-demand cortical hubs. However, causality cannot be inferred from cross-sectional data. Longitudinal validation is essential to determine whether changes in SMA connectivity precede cognitive decline and whether alleviating respiratory symptoms can reverse network disintegration. SMA_L’DC emerges as a promising biomarker for early risk stratification in COPD.

## Data Availability

The raw data supporting the conclusions of this article will be made available by the authors, without undue reservation.

## Footnotes

1
http://surfer.nmr.mgh.harvard.edu/

2
https://fsl.fmrib.ox.ac.uk/fsl/fslwiki

3
http://www.nitrc.org/projects/gretna/

## Glossary

AAL: Automated Anatomical Labeling
ABG: Arterial Blood Gas
ANCOVA: Analysis of Covariance
BCa: Bias-Corrected and Accelerated
BMI: Body Mass Index
CIs: Confidence Intervals
COPD: Chronic Obstructive Pulmonary Disease
COPD-SCV: COPD-related Subclinical Cognitive Vulnerability
Cp: Clustering Coefficient
CSF: Cerebrospinal Fluid
DC: Degree Centrality
DTI: Diffusion Tensor Imaging
E_glob: Global Efficiency
FA: Fractional Anisotropy
FEV₁: Forced Expiratory Volume in One Second
FVC: Forced Vital Capacity
FWE: Family-Wise Error
IOG: Inferior Occipital Gyrus
GM: Gray Matter
GOLD: Global Initiative for Chronic Obstructive Lung Disease
HC: Healthy Control
Lp: Path Length
MCI: Mild Cognitive Impairment
MMSE: Mini-Mental State Examination
MoCA: Montreal Cognitive Assessment
MNI: Montreal Neurological Institute
MRI: Magnetic Resonance Imaging
NCs: Normal Controls
PET: Positron Emissions Tomography
RD: Radial Diffusivity
SFG: Superior Frontal Gyrus
SGRQ: St. George’s Respiratory Questionnaire
SMA: Supplementary Motor Area
SMA_L: Left Supplementary Motor Area
SMA_L’ DC: the DC of the Left Supplementary Motor Area
SPM: Statistical Parametric Mapping
STG: Superior Temporal Gyrus
WM: White Matter
